# Impact of Inoculations with Indigenous *Bradyrhizobium diazoefficiens* Isolates on Productivity and Competition with Indigenous Bradyrhizobia in Adzuki Bean (*Vigna angularis*)

**DOI:** 10.1264/jsme2.ME24069

**Published:** 2025-03-22

**Authors:** Sokichi Shiro, Ryu Makihara, Shota Nakao, Masayuki Kadowaki, Yuichi Saeki

**Affiliations:** 1 Institute of Agricultural and Life Sciences, Academic Assembly, Shimane University, 1060 Nishikawatsu-cho Matsue, Shimane 690–8504, Japan; 2 Major in Agricultural and Life Sciences, Graduate School of Natural Science and Technology, Shimane University, 1060 Nishikawatsu-cho Matsue, Shimane 690–8504, Japan; 3 Department of Agricultural and Forest Sciences, Faculty of Life and Environmental Sciences, Shimane University, 1060 Nishikawatsu-cho Matsue, Shimane 690–8504, Japan; 4 Department of Biochemistry and Applied Biosciences, Faculty of Agriculture, University of Miyazaki, 1–1 Gakuen Kibanadai-nishi, Miyazaki 889–2192, Japan

**Keywords:** adzuki bean, nitrogen fixation, inoculation effect, competition

## Abstract

We herein exami­ned the inoculation effects of indigenous *Bradyrhizobium diazoefficiens* isolates on the growth and yield of adzuki beans and their competition with other bradyrhizobia using pot and field experiments. In the pot experiment, shoot nitrogen content was significantly higher following inoculations with AMP1 and Bd (a mixture of AN9 and AN20) than with the control. Furthermore, a correlation was observed between shoot nitrogen content and shoot dry weight. In the field experiment, the inoculating isolates did not significantly affect growth or yield. However, an interaction effect was observed in pod numbers and yield, suggesting that the effects of inoculation varied depending on the cultivar and inoculating isolate. In the correlation ana­lysis, pod number correlated with node number and nodule number. Similarly, yield correlated with shoot length, node number, nodule number, and pod number. Regarding competition between inoculated isolates and other strains, *B. elkanii* was dominant in pot and field experiments. To enhance the yield of adzuki bean through inoculations, it is necessary to overcome competition from indigenous *B. elkanii* and increase the occupancy rate of *B. diazoefficiens* isolates.

Adzuki bean (*Vigna angularis*) is predominantly produced in East Asian countries, including Japan, Korea, and China, and also in non-East Asian countries, such as Canada, for export to Japan. In Japan, the planting area and total harvest of adzuki bean were 21,300 to 32,000 ha and 29,500 to 76,800 tons, respectively, between 2014 and 2023 (MAFF, 2024). In China, the planting area and total harvest of adzuki bean were 134,700 to 221,100 ha and 208,000 to 360,000 tons, respectively, between 2016 and 2021 (Xiao, 2024). Although proper fertilizer management and the relationship between the genotype and cultivation environment have both been considered in order to improve adzuki bean productivity (Yin *et al.*, 2019; Hu *et al.*, 2022), the further utilization of symbiotic nitrogen fixation is needed to achieve sustainable adzuki bean production.

Adzuki bean, a legume, symbiotically fixes nitrogen with root nodule bacteria (rhizobia), including the genera *Bradyrhizobium*, *Rhizobium*, and *Sinorhizobium* (Fujihara *et al.*, 2006). Since adzuki beans have high nitrogen requirements and reliance on nitrogen fixation over soil nitrogen (Kimura *et al.*, 2004), symbiotic nitrogen fixation by rhizobia with a superior nitrogen-fixing ability is needed to improve productivity. However, the nitrogen-fixing ability of indigenous rhizobia strains is often poor (Vlassak *et al.*, 1997; Mason *et al.*, 2017, 2021); therefore, inoculations with selected superior nitrogen-fixing root nodule bacteria continue to be attempted. Delić *et al.* (2010) conducted inoculation tests on adzuki beans in cultivation pots with four strains of *Bradyrhizobium japonicum* and three strains of *Bradyrhizobium* spp. isolated from Serbian soil, and showed that shoot nitrogen content was higher with the two strains of *B. japonicum* than with the non-inoculated treatment, indicating the high nitrogen-fixing ability of these two strains. In addition, in the case of mung bean (*Vigna radiata*), cowpea (*Vigna unguiculata*), and black gram (*Vigna mungo*), which belong to the same genus of *Vigna* as adzuki bean, inoculations with *Bradyrhizobium* strains and co-inoculations with *Bradyrhizobium* strains and arbuscular mycorrhizal fungi were found to improve nitrogen fixation efficiency and productivity for shoot growth and seed yield (Christopher *et al.*, 2018; Ayalew and Yoseph, 2020; Pereira *et al.*, 2020; Gough *et al.*, 2021). Regarding the nitrogen-fixing ability of soybean-nodulating bradyrhizobia, a *Bradyrhizobium* strain with an uptake hydrogenase (Hup) system has been reported. This system absorbs hydrogen generated by nitrogenase in soybean nodule symbiosis. A Hup^+^ strain was shown to have higher nitrogen fixation efficiency in the nodules than a Hup^–^ strain (Evans *et al.*, 1987; Masuda *et al.*, 2016). Furthermore, *B. diazoefficiens* USDA110^T^ (formerly *Bradyrhizobium japonicum* USDA110^T^, Delamuta *et al.*, 2013) is reportedly a Hup^+^ strain (Saeki *et al.*, 2004). *B. diazoefficiens* USDA110^T^ is globally used in soybean research because of its high symbiotic efficiency and nitrogen-fixing ability (Champion *et al.*, 1992; Soe *et al.*, 2012; Chibeba *et al.*, 2017; Mason *et al.*, 2021). It is a valuable strain for soybean cultivation due to its role as a useful rhizobia (Shiro and Saeki, 2022). The Hup system’s effectiveness in enhancing legume crop productivity has been demonstrated for cowpea and mung bean (Drevon *et al.*, 1987). In addition, *B. diazoefficiens* USDA110^T^ infects adzuki beans to form nodules, and indigenous strains similar to *B. diazoefficiens* USDA110^T^ have been detected in Japanese soil (Shiro *et al.*, 2023). Therefore, the inoculation of adzuki beans with a *B. diazoefficiens* indigenous strain carrying genes for the Hup system associated with superior nitrogen-fixing ability may increase productivity. However, since indigenous strains are also present in the soil of adzuki bean cultivation fields, the degree to which inoculated strains establish a symbiotic relationship with adzuki beans and their competitiveness need to be exami­ned.

In the present study, adzuki bean-nodulating *B. diazoefficiens* indigenous strains carrying genes of the Hup system related to nitrogen-fixing ability were isolated from soil in Shimane Prefecture, Japan (located on the Sea of Japan in western Honshu), and the effects of inoculations with these strains on the growth and productivity of adzuki beans were exami­ned. Furthermore, we attempted to clarify their availability as a superior inoculant in adzuki bean cultivation. Their competition with indigenous strains, such as *B. japonicum* and *B. elkanii*, and their impact on symbiosis were also assessed.

## Materials and Methods

### Inoculation cultivation examination using planting pots

To examine the effects of *B. diazoefficiens* isolates on adzuki bean growth and isolate competition, we conducted an inoculation cultivation test using these isolates. Five isolates, AMP1, AN1, AN2, AN9, and AN20, from soil in Shimane Prefecture, Japan were used as the test strains. AMP1 was isolated from the root nodules of adzuki bean cultivated in soil collected in Matsue (35.4872 °N, 133.0696 °E) (Shiro *et al.*, 2023). AN1, AN2, AN9, and AN20 were isolated from the root nodules of adzuki bean cultivated in soil collected in Izumo (35.4307 °N, 132.8334 °E). A restriction fragment length polymorphism (RFLP) ana­lysis of the 16S-23S rRNA gene internal transcribed spacer (ITS) region revealed that AMP1, AN9, and AN20 were strains with similar RFLP patterns to *B. diazoefficiens* USDA 110^T^, while AN1 and AN2 were strains with similar RFLP patterns to *B. elkanii* USDA 31. In addition, for AMP1, AN9, and AN20, the *hup* gene associated with the Hup system was amplified by PCR, and these strains showed similar characteristics to *B. diazoefficiens* USDA 110^T^. These isolates were grown in Yeast-Mannitol (YM) liquid medium (K_2_HPO_4_ 0.5 g, MgSO_4_·7H_2_O 0.2 g, NaCl 0.1 g, Yeast Extract 0.4‍ ‍g, and D-(–)-Mannitol 10 g, up to 1 L distilled water, pH 6.8, autoclaved at 121°C for 20‍ ‍min) (Vincent, 1970) at 28°C for 7 days with shaking and then diluted to 10^6^‍ ‍cells‍ ‍mL^–1^ with sterile distilled water for use as an inoculum. Adzuki beans were cultivated in 1/5,000 a Wagener pots filled with a vermiculite and culture soil (mainly andosol) mixture (the mixing ratio of vermiculite to culture soil was 40 L to 20 kg) using two test cultivars, Tanba-dainagon and Toyomi-dainagon. Vermiculite and culture soil were commercial products that were sterilized during the manufacturing process. The fertilizers used were ammonium sulfate, superphosphate of lime, and potassium chloride, with N, P, and K levels of 3, 10, and 10‍ ‍kg 10 a^–1^, respectively. Fertilizer application levels were selected based on the website of the Japan Pluses Association (https://www.mame.or.jp/saibai/azu_souron.html).

Adzuki bean seeds were sterilized using a 70% ethanol soak for 30‍ ‍s and a 2.5% sodium hypochlorite solution (0.25% available chlorine) for 3‍ ‍min, and were then rinsed with sterile distilled water. Seeds were sown on July 23, 2019, with various inoculated treatments: Be (a mix of AN1 and AN2), Bd (a mix of AN9 and AN20), AMP1 (alone), BeBd (a mix of Be and Bd), BeAMP1 (a‍ ‍mix of Be and AMP1), and a non-inoculated control. Each seed was inoculated with 1‍ ‍mL of a diluted bacterial culture. The pot experiment was conducted as a two-factorial design: cultivar × inoculation, in three replicates. Pots were set up in an outdoor environment. Each pot contained two plants. Adzuki beans were grown outdoors for 7‍ ‍weeks, with irrigation as needed. After 7‍ ‍weeks, shoot length, node number, shoot dry weight, the SPAD value, nodule number, nodule fresh weight, and shoot nitrogen content were assessed. The SPAD value was measured using the SPAD-502 Chlorophyll Meter (KONICA MINOLTA) and read three times per leaf at the central part of each leaf in developing second leaves. The shoot was dried at 80°C for 72‍ ‍h using a drying machine. Shoot nitrogen content was measured using a highly sensitive nitrogen carbon analyzer (SUMIGRAPH^®^ NC-90A; Sumika Chemical Analysis Service).

To assess the inoculum occupancy rate in the BeBd and BeAMP1 treatments, approximately 24 nodules per pot were randomly selected and soaked in 70% ethanol for 3‍ ‍min and a 2.5% sodium hypochlorite solution (0.25% available chlorine) for 30‍ ‍min to surface sterilize the nodules. The number of nodules analyzed was selected based on previous studies (Shiro *et al.*, 2012, 2016; Shiro and Saeki, 2022). Post-sterilization, the nodules were rinsed with sterile distilled water. The total DNA of bradyrhizobia was extracted from these nodules, and PCR was performed targeting the *hupL* (bll6941) and *hupS* (bll6942) genes related to the Hup system. The occupancy rates of Bd, AMP1, and Be were evaluated by the presence of these gene amplifications. The specifics of the DNA extraction and PCR methods are outlined below.

### Inoculation cultivation test under field conditions

To assess the inoculation effects of *B. diazoefficiens* isolates on adzuki bean growth, yield, and competition between the inoculum and indigenous bradyrhizobia, we conducted an inoculation cultivation test using these isolates in an experimental field. Three isolates, AMP1, AMS5, and AN9, which were isolated from soil in different areas of Shimane Prefecture, Japan, were used as the test strains. AMS5 was isolated from the root nodules of adzuki beans cultivated in soil collected in Masuda (34.6793 °N, 131.9305 °E) (Shiro *et al.*, 2023). This strain, as well as AMP1 and AN9, possesses similar RFLP patterns for the 16S-23S rRNA gene ITS region and *hup* gene to *B. diazoefficiens* USDA 110^T^. Furthermore, AMS5 exhibited similar performance to AMP1 and AN9 for shoot growth in inoculation tests on adzuki bean (data not shown). These isolates were cultured in YM liquid medium at 28°C for 7 days with shaking, and were then diluted to 10^6^‍ ‍cells‍ ‍mL^–1^ with sterile distilled water for use as an inoculum. Four cultivars, Iwate-dainagon, Hokuto-dainagon, Takara-shozu, and Tanba-dainagon, were used as the test cultivars. The field trial was conducted at the Agricultural Science Section, Education and Research Center for Biological Resources, Faculty of Life and Environmental Science, Shimane University, Japan (35.5152 °N, 133.1100 °E). The experimental field featured gray lowland soil (paddy conversion field), with soil properties shown in Table 1. The fertilizers used were ammonium sulfate, superphosphate of lime, and potassium chloride, with N, P, and K levels of 3, 10, and 10‍ ‍kg 10 a^–1^, respectively. Fertilizer application levels were selected based on the website of‍ ‍the Japan Pluses Association (https://www.mame.or.jp/saibai/azu_souron.html). Typical fertilizer levels for adzuki bean are 3 to 4, 10 to 18, and 8 to 12‍ ‍kg 10 a^–1^ of N, P, and K, respectively. The amount of fertilizer applied varies depending on the regional climate and soil fertility of the field. In the present study, fertilizer application levels appropriate for a warm region were selected based on these values. To adjust soil pH, magnesium lime was applied at a dose of 100‍ ‍kg 10 a^–1^. Seeds of Iwate-dainagon, Hokuto-dainagon, and Takara-shozu were sown on June 16, 2021, and those of Tanba-dainagon on July 17, 2021. Sowing was conducted by hand and was not treated for surface sterilization. Inoculations with the test strains were performed concurrently with seeding, with inoculated and non-inoculated treatments both being designated. The inoculated treatment involved the addition of 1‍ ‍mL of the prepared bacterial suspension per seed. The planting density was 15.2 plants m^–2^. The area where Iwate-dainagon, Hokuto-dainagon, and Takara-shozu was cultivated was 96 m^2^ and the area where Tanba-dainagon was cultivated was 18 m^2^. This field experiment was conducted as a two-factorial design: cultivar × inoculation, in a randomized block design with three replications.

Growth and yield investigations for Iwate-dainagon, Hokuto-dainagon, and Takara-shozu were conducted on September 1, 2021, at the end of the flowering period, and October 14, during the full ripened stage, respectively. Regarding Tanba-dainagon, these investigations were conducted on September 16, 2021, during the flowering period, and November 16, during the full ripened stage, respectively. To obtain fresh nodules for an ana­lysis of the occupancy of infected bradyrhizobia, growth investigations were conducted before the seedling fertilization stage. The growth investigation included shoot length, node number, shoot dry weight, and nodule number, while the yield investigation included pod number, seed number, 100-seed weight, and yield. Shoot dry weight was measured after drying plants at 70°C for at least 72 h. In the yield investigation, only pods were collected at harvest and air-dried. Nodules attached to the roots were collected during the growth investigation, counted, and surface sterilized by soaking in 70% ethanol for 3‍ ‍min and a 2.5% sodium hypochlorite solution (0.25% available chlorine) for 30‍ ‍min, followed by rinsing with sterile distilled water. Twenty-four nodules per treatment were randomly selected for a subsequent ana­lysis. The total DNA of bradyrhizobia was extracted from these nodules, and PCR targeting the 16S-23S rRNA gene ITS region and *hupL* and *hupS* genes was performed. The occupancy rates of the inoculum and indigenous bradyrhizobia were exami­ned by the RFLP pattern of ITS and the presence of *hupL* and *hupS* gene amplification. Since the genes of the Hup system related to nitrogen-fixing ability were possessed by strains of *B. diazoefficiens*, including the test strains, the isolates that showed RFLP patterns similar to *B. diazoefficiens* USDA 110^T^ were investigated. The specifics of the DNA extraction and PCR methods are outlined below.

### Analysis of adzuki bean-nodulated bradyrhizobia

Total DNA for the PCR template was directly extracted from a nodule as previously described (Shiro *et al.*, 2016) using the method reported by Hiraishi *et al.* (1995). Each nodule was homogenized in 50‍ ‍μL of BL buffer (40‍ ‍mM Tris-HCl, 1% Tween 20, 0.5% Nonidet P-40, and 1‍ ‍mM EDTA, pH 8.0), 40‍ ‍μL of sterile distilled water, and 10‍ ‍μL of 1‍ ‍mg mL^–1^ proteinase K, and was then incubated at 60°C for 20‍ ‍min and 95°C for 5‍ ‍min. Post-centrifugation, the supernatant was collected and used for PCR amplification of the 16S-23S rRNA gene ITS region and *hupL* (bll6941) and *hupS* (bll6942) genes, which are related to the Hup system of nodulated adzuki bean bradyrhizobia.

PCR for the 16S-23S rRNA gene ITS region was conducted using Tks Gflex^TM^ DNA Polymerase (Takara Bio). The primer set used for ITS region amplification was BraITS-F (5′-GACTGGGGTGAAGTCGTAAC-3′) and BraITS-R (5′-ACGTCCTTCATCGCC TC-3′) (Saeki *et al.*, 2006). One microliter of the template was used as the template for PCR. The PCR cycle included an initial 94°C for 3‍ ‍min; 30 cycles at 98°C for 10‍ ‍s, 60°C for 15‍ ‍s, and 68°C for 30 s; and a final cycle at 68°C for 2‍ ‍min. In the RFLP ana­lysis of the ITS region, the PCR product was digested with *Msp*I (Takara Bio) at 37°C for 16 h. Fragments were separated by electrophoresis using a 3% agarose gel and visualized with ethidium bromide. As reference strains to identify the type of nodulated adzuki bean bradyrhizobia, *B. japonicum* USDA 4, 6^T^, 38, 115, 123, 124, and 135, *B. diazoefficiens* USDA 110^T^, and *B. elkanii* USDA 31, 46, and 94 were used (Saeki *et al.*, 2004; Shiro *et al.*, 2023). These reference strains were provided by the Laboratory of Soil Science and Plant Nutrition, Faculty of Agriculture, University of Miyazaki. The restriction enzyme-digested products of root nodule-derived ana­lysis samples and the reference strain were electrophoresed simultaneously on the same agarose gel. The reference strains were used for electrophoresis each time. The fragment sizes on the electrophoresed gel were measured with a 50 bp DNA ladder marker (FastGene). All reproducible fragments longer than 50 bp were used to identify similarities and some irreproducible fragments were excluded (Shiro *et al.*, 2023). The isolates that showed similar RFLP patterns to *B. japonicum* USDA 4, 6^T^, 38, 115, 123, 124, and 135, *B. diazoefficiens* USDA 110^T^, and *B. elkanii* USDA 31, 46, and 94 were designated as Bj4, 6, 38, 115, 123, 124, and 135, Bd110, and Be31, 46, and 94, respectively.

PCR for the *hupL* and *hupS* genes was conducted using either EmeraldAmp^®^ MAX PCR Master Mix (Takara Bio) or TaKaRa Ex Premier^TM^ DNA Polymerase Dye Plus (Takara Bio). The primer set used for *hupL* and *hupS* gene amplification included hupS-F261 (5′-TCGAACAGGCGTTGTAAGTG-3′), hupS-R830 (5′-TCGACTACGACGACGACACCATC-3′), hupL-F962 (5′-TCGGGCAGATAGACCATTTC-3′), and hupL-R1632 (5′-GGGATCGAAGTGATCCTGAA-3′) (Shiro and Saeki, 2022). One microliter of the template was used as the template for PCR. The PCR cycle with EmeraldAmp^®^ MAX PCR Master Mix involved an initial 94°C for 5‍ ‍min; 30 cycles at 94°C for 30‍ ‍s, 60°C for 30‍ ‍s, and 72°C for 60 s; and a final cycle at 72°C for 5‍ ‍min. The PCR cycle with TaKaRa Ex Premier^TM^ DNA Polymerase Dye Plus involved an initial 94°C for 3‍ ‍min; 30 cycles at 98°C for 10‍ ‍s, 58°C for 15‍ ‍s, and 68°C for 30 s; and a final cycle at 68°C for 2‍ ‍min. Fragments were separated by electrophoresis using a 2% agarose gel and visualized with ethidium bromide. Samples in which the *hupL* and *hupS* genes were both amplified were defined as Hup^+^ strains, while those in which either one or neither gene was amplified were defined as Hup^–^ strains.

### Statistical ana­lysis

Statistical ana­lyses were performed using R version 4.2.2 (R Core Team, 2022). To assess the effects of *B. diazoefficiens* isolate inoculations on the growth yield and occupancy rate of adzuki bean-nodulated bradyrhizobia and the cultivar, a two-way ANOVA was conducted using anovakun version 4.8.6 (Iseki, 2021), an analysis of variance function in R. Tukey’s HSD test was also performed using the multcomp package in R. The significance of relationships between each investigation item was tested using the cor.test function in R.

## Results

### Inoculation cultivation test using planting pots

**Fig. 1.**
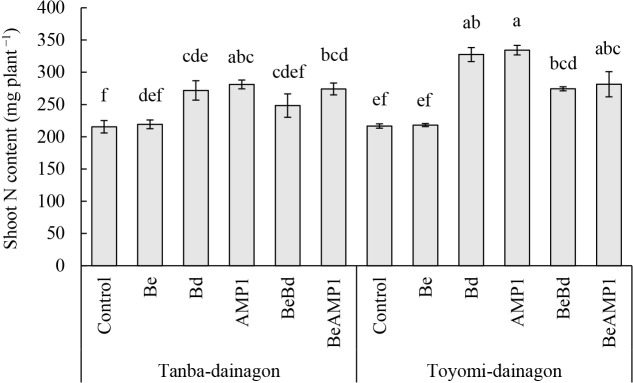
Impact of adzuki bean cultivars and inoculations with *Bradyrhizobium diazoefficiens* isolates on shoot N content. Values represent the mean±standard error (*n*=3). The significance of differences between different letters was assessed by Tukey’s HSD test (*P*<0.05).

The results of the cultivation experiment involving single or mixed inoculations of isolates are shown in Table 2. In the variance ana­lysis, significant differences were observed in all investigated items, except for nodule fresh weight for the cultivars and nodule fresh weight and shoot nitrogen content for the treatments. In the treatment, inoculations with AMP1 and Bd generally showed higher shoot lengths, node numbers, shoot dry weights, and shoot nitrogen contents than the other treatments. Shoot dry weight and shoot nitrogen content for the Be single and mixed inoculations were slightly lower than those for the AMP1 and Bd inoculations, but were higher than the control values. An interaction was detected for shoot nitrogen content (*F* [5, 24]=2.798, *P*=0.0396), prompting a multiple comparison test across all counties, with the results obtained shown in Fig. 1. Both cultivars exhibited slightly higher values with the AMP1 or Bd inoculation, with Toyomi-dainagon showing higher values than Tanba-dainagon, indicating cultivar-specific responses.

The relationships between each investigation item in the pot cultivation test are shown in Table 3. Correlations were observed between shoot length and node number or shoot dry weight, between node number and shoot dry weight or shoot nitrogen content, and between shoot dry weight and shoot nitrogen content. Furthermore, shoot length was associated with shoot nitrogen content, and SPAD with nodule fresh weight.

Regarding the mixed inoculated treatments of BeBd and BeAMP1, we analyzed the occupancy of infected test strains (Table 4). In Tanba-dainagon, BeBd showed 66.7% of Be and 33.3% of Bd, while BeAMP1 showed 58.3% of Be and 41.7% of AMP1. In Toyomi-dainagon, BeBd showed 54.2% of Be and 45.8% of Bd, while BeAMP1 showed 66.7% of Be and 33.3% of AMP1. Both cultivars exhibited a higher occupancy rate of Be than Bd or AMP1.

### Inoculation cultivation test under field conditions

The effects of *B. diazoefficiens* isolate inoculations on adzuki bean growth and yield are shown in Table 6. An ana­lysis of variance showed significant differences in all investigated items among cultivars, but only in node numbers among inoculation treatments. Among the cultivars, Tanba-dainagon displayed the highest values in all growth investigation items, with significantly higher values for shoot length, node number, and shoot dry weight than the other three cultivars and significantly higher values for nodule number than Hokuto-dainagon and Takara-shozu. In the yield investigation, Iwate-dainagon had the highest values for pod number and seed number, while Tanba-dainagon had the highest values for 100-seed weight and yield. Notably, Iwate-dainagon had a significantly higher pod number than the three other cultivars, Iwate-dainagon and Tanba-dainagon had significantly higher seed numbers than Hokuto-dainagon, Tanba-dainagon had a significantly higher 100-seed weight than the three other cultivars, and Tanba-dainagon had a significantly higher yield than Takara-shozu. Regarding differences among treatments, Tukey’s HSD test on node number, which showed significant differences in the ana­lysis of variance, revealed no significant differences among treatments, and the inoculation with AMP1 yielded slightly higher values than the other treatments. The AMP1 inoculation yielded slightly higher values than the other treatments for all investigated items, except nodule number.

**Fig. 2.**
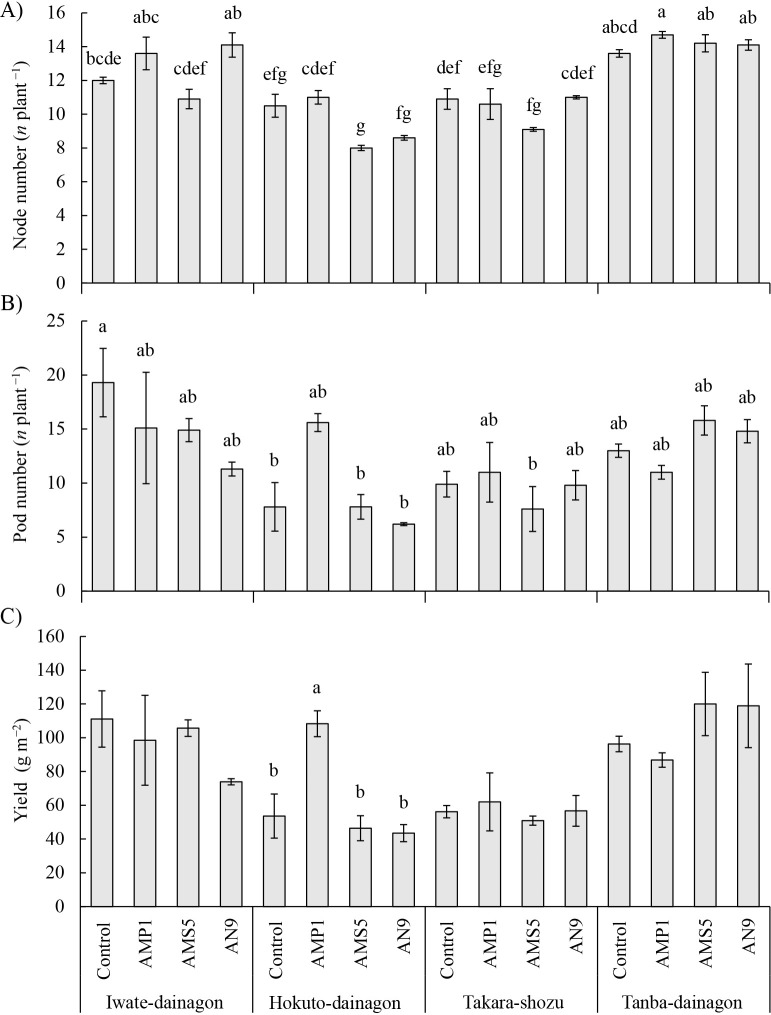
Impact of adzuki bean cultivars and inoculations with *Bradyrhizobium diazoefficiens* isolates. A, B, and C represent node number, pod number, and yield, respectively. Values represent the mean±standard error (*n*=3). The significance of differences between different letters was assessed by Tukey’s HSD test (*P*<0.05). Regarding yield, significant differences were only detected within Hokuto-dainagon; therefore, the results of Tukey’s HSD test (*P*<0.05) are denoted in different letters.

Node number (*F* [9, 32]=3.297, *P*=0.0060), pod number (*F* [9, 32]=2.485, *P*=0.0280), and yield (*F* [9, 32]=2.603, *P*=0.0223) showed significant interaction differences; therefore, Tukey’s HSD test was conducted for the 16 groups, with the results obtained being shown in Fig. 2. Regarding node number, Iwate-dainagon generally exhibited higher values with the AMP1 and AN9 inoculations, while Hokuto-dainagon and Tanba-dainagon showed higher values with the AMP1 inoculation than with the control. Takara-shozu typically displayed lower values with all inoculated treatments than with the control (Fig. 2A). Hokuto-dainagon and Takara-shozu generally had higher pod numbers with the AMP1 inoculation and Tanba-dainagon had higher pod numbers with the AMS5 and AN9 inoculations than with the control, while Iwate-dainagon typically had lower pod numbers with all inoculated treatments than with the control (Fig. 2B). Similar results to those for pod numbers were observed for yield, particularly for Hokuto-dainagon, which had significantly higher values than the other treatments due to the AMP1 inoculation (Fig. 2C).

The relationships among data obtained from the growth and yield investigations are shown in Table 7. Correlations were observed between shoot length and node number, shoot dry weight, nodule number, 100-seed weight, and yield; between node number and shoot dry weight, nodule number, pod number, and yield; between shoot dry weight and nodule number, 100-seed weight, and yield; between nodule number and pod number, seed number, and yield; between pod number and seed number and yield; between seed number and yield; and between 100-seed weight and yield.

**Fig. 3.**
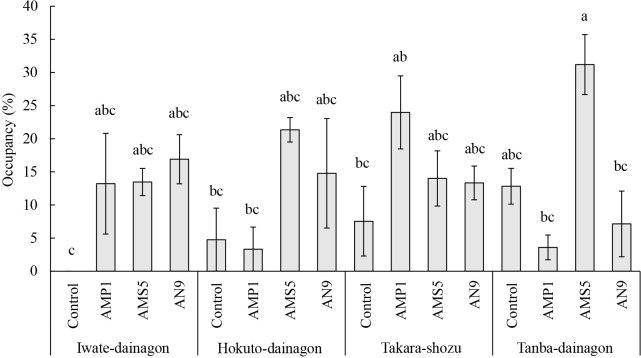
Occupancy rate (%) of the Hup^+^ strain of Bd110. Values represent the mean±standard error (*n*=3). The significance of differences between different letters was assessed by Tukey’s HSD test (*P*<0.05).

The occupancy of inoculated *B. diazoefficiens* isolates and indigenous adzuki bean-nodulated bradyrhizobia is shown in Table 8. A variance ana­lysis revealed no significant differences in any investigated items among cultivars; however, significant differences were found among inoculation treatments in the Bj6 and Hup^+^ strains of Bd110. Notably, the AMS5 inoculation showed a significantly higher occupancy rate of the Hup^+^ strain than the control, and the AMP1 and AN9 inoculations also showed a slightly higher occupancy rate than the control. However, an interaction between cultivar and inoculation treatment was detected for the occupancy rate of the Hup^+^ strain (*F* [9, 32]=3.279, *P*=0.0062), prompting Tukey’s HSD test on 16 groups, with the results obtained being shown in Fig. 3. In comparisons with the control, the occupancy rate of the Hup^+^ strain was slightly higher with the AN9 inoculation in Iwate-dainagon, the AMS5 inoculation in Hokuto-dainagon and Tanba-dainagon, and the AMP1 inoculation in Takara-shozu.

## Discussion

In the present study, we exami­ned the effects of inoculations with various isolates from the adzuki bean nodules of *B. diazoefficiens* carrying the *hupL* and *hupS* genes of the Hup system, which are related to nitrogen-fixing ability, on the growth and yield of adzuki bean. We also investigated how the occupancy of adzuki bean-nodulated bradyrhizobia was affected by inoculations with these isolates and evaluated their competitiveness with indigenous bradyrhizobial strains. In a pot cultivation experiment, we investigated the effects of a single or mixed inoculation of isolates with competing isolates on the growth of adzuki bean and the occupancy rate of infected bradyrhizobia. The results of the growth investigation indicated that in terms of shoot length and shoot dry weight, the AMP1 and Bd inoculations generally outperformed the Be single inoculation and the control (Table 2). Moreover, the shoot nitrogen contents of the AMP1 and Bd inoculations were significantly higher than those of the Be single inoculation and the control (Table 2). The values for the BeBd and BeAMP1 mixed inoculations were not similar to those for the AMP1 and Bd inoculations (Table 2). A correlation ana­lysis revealed a correlation between shoot nitrogen content and shoot dry weight (Table 3), suggesting that the AMP1 and Bd inoculations obtained more nitrogen through nitrogen fixation, thereby promoting shoot growth. The mixed inoculation of BeBd and BeAMP1 was not as effective as the single inoculation, presumably because AMP1 and Bd competed with Be, resulting in an inability to maintain a high occupancy rate (Table 4).

Meteorological conditions during the cultivation period in the field cultivation experiment are shown in Table 5. Temperatures exceeded the yearly average, except for August. Precipitation was approximately 2.1-fold higher in July and approximately 4-fold higher in August than in the average year. Sunshine duration surpassed the yearly average, except for August and September. Although the cultivation period, growing environment, and soil conditions were different, the growth conditions of adzuki beans in the present study (Table 6) were considered to be poorer than in previous cultivation tests (Yoneda *et al.*, 1973; Sato *et al.*, 1975; Shimada *et al.*, 1997; Ushio *et al.*, 2013). Adzuki bean is sensitive to cool weather and moisture damage, with flooding causing poor germination and delays in flowering and maturity (Goto *et al.*, 1985). In August 2021, when the investigation was conducted, rainfall and sunshine duration were both lower than in an average year (Table 5). Therefore, Iwate-dainagon, Hokuto-dainagon, and Takara-shozu, sown in June, may have been affected by weather during the vegetative and reproductive stages, while Tanba-dainagon, sown in July, may have been affected during the vegetative stage, leading to suppressed growth and subsequent seed production. The inoculation effects of *B. diazoefficiens* isolates were not significant based on the results of growth and yield investigations (Table 6). However, interaction effects were detected in the ana­lysis of variance for node number, pod number, and yield (Fig. 2). The yield of Hokuto-dainagon was significantly higher with the AMP1 inoculation than with the control (Fig. 2C), suggesting differences in the symbiotic performance of inoculum strains among cultivars or differences in the traits of cultivars, such as nitrogen usage efficiency. Additionally, a correlation ana­lysis among the investigated items, using data from the growth and yield investigations, revealed that shoot length, node number, and nodule number correlated with pod number and yield (Table 7). A previous study reported that shoot length, node number, and branch number correlated with yield in adzuki bean (Hu *et al.*, 2022), indicating that enhancing shoot growth, including increases in node and branch numbers, was associated with greater yield. In the present study, besides these items, nodule number correlated with yield (Table 7), suggesting that an increase in nodule number improved shoot growth, contributing to greater yield. The increase in nodule number may have been affected by the inoculation with *B. diazoefficiens* isolates. However, no significant inoculation effect of *B. diazoefficiens* isolates was detected (Table 6). A possible reason for this is that Be31, which exhibits a similar RFLP pattern to *B. elkanii* USDA 31, was dominant in all cultivars and treatments, and the occupancy of Hup^+^ strains carrying the *hupL* and *hupS* genes was low, ranging between 3.3 and 31.2% in all test strain-inoculated treatments (Table 8). Since the results of the pot cultivation experiment suggest that inoculations with the Hup^+^ strains, AMP1 and Bd (AN9 and AN20), provided more nitrogen through nitrogen fixation (Table 2), it may be beneficial to develop a technique to increase the occupancy of Hup^+^ strains. However, since the occupancy of the Hup^+^ strain differed among adzuki bean cultivars (Fig. 3), compatibility between the adzuki bean and inoculum strain also needs to be exami­ned.

In soybean, which is symbiotic with the root nodule bacteria of the same genus of *Bradyrhizobium* as adzuki beans, the presence of the *Rj* gene, which restricts symbiosis with certain bradyrhizobial strains, has been reported (Hayashi *et al.*, 2012). In addition, the *Rj* gene has been shown to affect the community structure of soybean-nodulated bradyrhizobia (Minami *et al.*, 2009; Shiro *et al.*, 2012). Although it remains unknown whether these genes are present in adzuki beans, since *Bradyrhizobium* strains have shown incompatibility with legumes of the genus *Vigna* (Nguyen *et al.*, 2017, 2020), a strong or weak degree of compatibility with bradyrhizobia may also exist in adzuki beans. However, Be31 was dominant in all adzuki bean cultivars used in the present study, which appears to be an important result. In soybean, *B. japonicum* is more competitive than *B. elkanii* with *Glycine max*, but less competitive with *G. soja* and *Macroptilium atropurpureum* (siratro) (Yuhashi *et al.*, 1995; Hungria *et al.*, 1998). When soybean roots were inoculated with *B. elkanii* USDA 31, USDA 76^T^, or USDA 94, localized swelling of the outer cortical cells of the roots was observed within 10 days after the inoculation, but not when roots were inoculated with *B. diazoefficiens* USDA110^T^ or USDA 122 (Yuhashi *et al.*, 1995). In adzuki beans, specific responses may be occurring in the roots that promote or are associated with compatibility for *B. elkanii* infection. In addition, the existence of some strains of *B. elkanii* has been reported to exert stronger symbiotic effects in soybean (Hungria *et al.*, 1998; Mason *et al.*, 2021; Leng *et al.*, 2023). Furthermore, *B. elkanii* USDA 94 has been reported to enhance the shoot length and dry weight of adzuki beans (Shiro *et al.*, 2023). The potential benefits of *B. elkanii* on the productivity of adzuki beans need to be investigated in detail.

Soil flooding is one method that is used to improve the occupancy of Hup^+^ strains, such as AMP1. *Bradyrhizobium*, particularly *B. diazoefficiens* USDA 110^T^, denitrifies under reductive soil conditions, converting nitrate ions to nitrogen gas (Sameshima-Saito *et al.*, 2006). The genes *napA*, *nirK*, *norCB*, and *nosZ* are involved in this process. USDA 110^T^, which carries the *nosZ* gene, dominates in alluvial and flooded soils (Shiina *et al.*, 2014; Saeki *et al.*, 2017). Additionally, *B. diazoefficiens*, which carries the *nosZ* gene, has been reported to have a higher chlo­rophyll content in the leaves and nitrogen content in the shoots of soybean than *B. japonicum* and *B. elkanii*, which do not carry this gene (Brignoli *et al.*, 2024). The strains AMP1, AMS5, AN9, and AN20 used in this study possess the *nosZ* gene (data not shown), suggesting that they fully denitrify. Therefore, inoculating the soil with these strains, followed by flooding and draining, and then cultivating adzuki beans may suppress infections by strains such as *B. elkanii* USDA 31, which lacks a full denitrification capability, and may also increase the occupancy of AMP1 and other inoculated strains with full denitrification capabilities. Based on this hypothesis, we conducted an experiment and obtained results for Hokuto-dainagon and Tanba-dainagon that revealed the slightly higher occupancy of AN9, which showed similar ITS region RFLP patterns to *B. diazoefficiens* USDA 110^T^, and higher occupancy than AN1, which showed similar ITS region RFLP patterns to *B. elkanii* USDA 31, due to soil flooding (data not shown). However, since occupancy by AN9 was less than 50%, the addition of other treatments to flooding may increase the occupancy of AN9.

In the present study, we were unable to obtain a clear inoculation effect of *B. diazoefficiens* isolates possessing the *hup* gene associated with nitrogen-fixing ability. However, we will continue to examine the issue of competitiveness with indigenous rhizobacteria, and also the potential benefits of *B. elkanii*, as we work to establish a more practical cultivation technique for improving adzuki bean productivity.

## Citation

Shiro, S., Makihara, R., Nakao, S., Kadowaki, M., and Saeki, Y. (2025) Impact of Inoculations with Indigenous *Bradyrhizobium diazoefficiens* Isolates on Productivity and Competition with Indigenous Bradyrhizobia in Adzuki Bean (*Vigna angularis*). *Microbes Environ ***40**: ME24069.

https://doi.org/10.1264/jsme2.ME24069

## Figures and Tables

**Table 1.** Soil properties of the experimental field.

pH (H_2_O)	EC (dS m^–1^)	NO_3_-N (mg kg^–1^)	NH_4_-N (mg kg^–1^)	P (mg kg^–1^)	K (mg kg^–1^)	Ca (mg kg^–1^)	Mg (mg kg^–1^)
6.09	0.076	1.07	16.9	371.4	252.2	1469	385.4

**Table 2.** Impact of cultivars and inoculations of isolates on the growth of adzuki beans in the pot cultivation examination.

Cultivar	Treatment	Shoot length (cm plant^–1^)	Node number (*n* plant^–1^)	SPAD	Shoot dry weight (g plant^–1^)	Nodule fresh weight (mg plant^–1^)	Shoot N content (mg plant^–1^)
Tanba-dainagon	Control	11.7±0.7	5.3±0.2	36.8±2.2	1.43±0.2	162.4±29.8	215.5±9.6
	Be	12.1±0.4	5.3±0.3	34.7±1.4	1.31±0.1	123.4±18.2	219.2±6.8
	Bd	11.5±0.7	5.2±0.3	35.3±1.3	1.46±0.1	132.9±67.1	271.9±15.1
	AMP1	12.1±0.8	5.2±0.2	37.3±5.1	1.46±0.1	216.9±59.6	281.1±6.8
	BeBd	10.8±1.3	5.0±0.3	37.3±1.5	1.30±0.2	311.9±49.3	248.3±18.2
	BeAMP1	12.6±0.6	5.2±0.2	39.9±0.3	1.47±0.1	334.8±39.1	274.1±9.2
Toyomi-dainagon	Control	9.8±0.4	4.0±0.0	31.6±1.7	1.64±0.2	198.9±64.7	216.7±3.4
	Be	9.9±0.2	4.2±0.2	31.5±0.8	1.92±0.1	230.2±51.9	218.2±2.4
	Bd	10.9±0.3	4.7±0.3	31.6±2.5	1.98±0.1	222.8±23.8	327.5±10.9
	AMP1	11.6±0.1	5.2±0.2	33.0±1.4	2.02±0.1	243.7±35.4	334.3±7.4
	BeBd	10.7±0.1	4.7±0.2	31.8±1.2	1.86±0.1	393.9±28.9	274.4±3.1
	BeAMP1	9.8±0.5	4.3±0.3	31.1±1.4	1.81±0.1	327.7±91.1	281.5±19.6
Tanba-dainagon		11.8±0.3	5.2±0.1	36.9±0.9	1.40±0.0	213.7±25.8	251.7±7.5
Toyomi-dainagon		10.4±0.2	4.5±0.1	31.8±0.6	1.87±0.1	269.5±25.0	275.4±11.8
	Control	10.7±0.6	4.7±0.3	34.2±1.7	1.53±0.1	180.6±32.9 bc	216.1±4.6 d
	Be	11.0±0.5	4.8±0.3	33.1±1.0	1.61±0.2	176.8±34.3 b	218.7±3.2 cd
	Bd	11.2±0.4	4.9±0.2	33.4±1.5	1.72±0.1	177.8±37.6 b	299.7±15.0 ab
	AMP1	11.9±0.4	5.2±0.1	35.2±2.6	1.74±0.1	230.3±31.6 ab	307.7±15.0 a
	BeBd	10.7±0.6	4.8±0.2	34.5±1.5	1.58±0.2	352.9±31.4 a	261.4±10.1 bc
	BeAMP1	11.2±0.7	4.8±0.3	35.5±2.1	1.64±0.1	331.3±44.4 ac	277.8±9.8 ab
ANOVA	Cultivar (C)	***	***	***	***	ns	***
	Treatment (T)	ns	ns	ns	ns	**	***
	C×T	ns	ns	ns	ns	ns	*

Values represent the mean±standard error (*n*=3). Asterisks denote significant differences at * *P*<0.05, ** *P*<0.01, and *** *P*<0.001. The significance of differences between different letters was assessed by Tukey’s HSD test (*P*<0.05).

**Table 3.** Correlation coefficients between investigation items in the pot cultivation examination.

	SL	NN	SPAD	SDW	NFW	SNC
SL	1.000					
NN	0.861*	1.000				
SPAD	0.377	0.237	1.000			
SDW	0.879*	0.862*	0.122	1.000		
NFW	–0.101	–0.042	0.688	–0.130	1.000	
SNC	0.741	0.817*	0.434	0.878*	0.254	1.000

Asterisks denote significant differences at * *P*<0.05. SL, NN, SDW, NFW, and SNC represent shoot length, node number, shoot dry weight, nodule fresh weight, and shoot nitrogen content, respectively.

**Table 4.** Occupancy rates (%) of Be, Bd, and AMP1 infecting adzuki bean.

	BeBd			BeAMP1	
	Be	Bd		Be	AMP1
Tanba-dainagon	66.7±8.3	33.3±8.3		58.3±8.3	41.7±8.3
Toyomi-dainagon	54.2±11.0	45.8±11.0		66.7±4.2	33.3±4.2
Average	60.4±6.8	39.6±6.8		62.5±4.6	37.5±4.6

Values represent the mean±standard error (*n*=3).

**Table 5.** Meteorological conditions during the cultivation period in 2021.

Month	Average (°C)			Precipitation (mm)	Sunshine duration (h)
	Temperature	Max. daily temperature	Min. daily temperature		
Jun.	22.2 (21.7)	26.7 (26.2)	18.8 (18.2)	158.0 (173.0)	164.9 (157.1)
Jul.	27.0 (25.8)	31.2 (29.8)	23.6 (22.8)	480.0 (234.1)	221.1 (168.6)
Aug.	26.5 (27.1)	30.6 (31.6)	23.6 (23.8)	517.5 (129.6)	142.7 (201.1)
Sept.	23.6 (22.9)	27.4 (27.1)	20.8 (19.6)	123.5 (204.1)	118.1 (146.2)
Oct.	18.1 (17.4)	23.1 (22.0)	14.2 (13.4)	86.0 (126.1)	181.8 (154.4)
Nov.	12.1 (12.0)	16.9 (16.5)	8.1 (8.0)	120.0 (121.6)	144.1 (113.8)

Meteorological data were sourced from the Japan Meteorological Agency website. The values in parentheses represent averages for the period of 1991–2020.

**Table 6.** Impact of cultivars and inoculations of isolates on the growth of adzuki beans in the field cultivation examination.

Cultivar	Treatment	Growth investigation					Yield investigation			
		Shoot length (cm plant^–1^)	Node number (*n* plant^–1^)	Shoot dry weight (g plant^–1^)	Nodule number (*n* plant^–1^)		Pod number (*n* plant^–1^)	Seed number (*n* plant^–1^)	100-seed weight (g)	Yield (g m^–2^)
Iwate-dainagon	Control	29.1±3.5	12.0±0.2	6.3±0.9	345.5±7.2		19.3±3.2	58.0±12.8	13.0±0.8	111.1±16.7
	AMP1	27.2±3.8	13.6±1.0	6.5±1.3	304.4±94.6		15.1±5.2	47.5±10.0	13.4±0.9	98.5±26.6
	AMS5	26.2±4.2	10.9±0.6	6.7±1.6	444.0±80.3		14.9±1.1	59.1±6.7	11.9±0.8	105.7±4.9
	AN9	25.6±1.5	14.1±0.7	7.5±1.4	330.3±40.0		11.3±0.6	43.7±0.9	11.1±0.1	73.9±1.8
Hokuto-dainagon	Control	23.4±2.4	10.5±0.7	9.8±0.9	134.3±20.9		7.8±2.2	23.1±5.3	15.2±0.2	53.6±13.1
	AMP1	22.5±2.1	11.0±0.4	9.5±2.4	173.0±39.3		15.6±0.8	45.8±3.9	15.6±0.2	108.3±7.7
	AMS5	18.9±1.1	8.0±0.2	5.8±1.2	115.6±27.1		7.8±1.1	22.1±3.2	13.8±0.5	46.4±7.4
	AN9	21.3±3.4	8.6±0.1	6.2±1.7	187.1±18.3		6.2±0.1	20.3±1.0	14.2±0.6	43.5±5.1
Takara-shozu	Control	18.6±0.7	10.9±0.6	5.7±1.3	196.9±61.9		9.9±1.2	40.8±2.1	9.0±0.2	56.2±3.7
	AMP1	20.9±1.7	10.6±0.9	6.9±0.9	181.2±38.7		11.0±2.8	44.1±12.1	9.2±0.1	62.0±17.2
	AMS5	19.6±0.7	9.1±0.1	4.6±0.5	148.1±25.6		7.6±2.1	37.7±3.6	9.0±0.4	50.9±2.7
	AN9	17.9±0.3	11.0±0.1	5.1±0.6	246.9±58.7		9.8±1.4	40.8±6.8	9.2±0.1	56.7±9.1
Tanba-dainagon	Control	67.3±9.3	13.6±0.2	17.0±2.2	350.9±41.7		13.0±0.6	43.1±2.0	14.8±1.2	96.3±4.6
	AMP1	67.6±9.9	14.7±0.2	17.8±0.1	428.8±77.0		11.0±0.6	35.5±2.2	16.1±0.3	86.8±4.3
	AMS5	67.7±4.8	14.2±0.5	16.0±1.1	548.2±85.1		15.8±1.4	46.6±6.9	16.9±1.4	120.0±18.8
	AN9	62.2±5.0	14.1±0.3	18.9±0.6	418.3±117.9		14.8±1.1	45.7±8.0	16.9±0.6	118.9±24.8
Iwate-dainagon		27.0±1.5 b	12.7±0.5 b	6.8±0.6 b	356.1±32.1 a		15.2±1.6 a	52.1±4.3 a	12.4±0.4 c	97.3±8.0 ab
Hokuto-dainagon		21.5±1.1 bc	9.5±0.4 c	7.8±0.9 b	152.5±14.6 b		9.4±1.2 b	27.8±3.6 b	14.7±0.3 b	63.0±8.8 ab
Takara-shozu		19.3±0.5 c	10.4±0.3 c	5.6±0.4 b	193.3±23.3 b		9.6±0.9 b	40.9±3.2 ab	9.1±0.1 d	56.5±4.4 b
Tanba-dainagon		66.2±3.3 a	14.2±0.2 a	17.4±0.6 a	436.6±42.0 a		13.7±0.7 ab	42.7±2.7 a	16.2±0.5 a	105.5±8.0 a
	Control	34.6±6.2	11.8±0.4	9.7±1.5	256.9±32.8		12.5±1.6	41.3±4.8	13.0±0.8	79.3±8.9
	AMP1	34.6±6.2	12.5±0.6	10.2±1.5	271.9±42.5		13.2±1.4	43.2±3.7	13.6±0.8	88.9±8.7
	AMS5	33.1±6.2	10.6±0.7	8.3±1.4	314.0±61.9		11.5±1.3	41.4±4.7	12.9±0.9	80.8±10.8
	AN9	31.8±5.5	12.0±0.7	9.4±1.7	295.7±39.6		10.5±1.0	37.6±3.9	12.9±0.9	73.3±10.3
ANOVA	Cultivar (C)	***	***	***	***		***	***	***	***
	Treatment (T)	ns	***	ns	ns		ns	ns	ns	ns
	C×T	ns	**	ns	ns		*	ns	ns	*

Values represent the mean±standard error (*n*=3). Asterisks denote significant differences at * *P*<0.05, ** *P*<0.01, and *** *P*<0.001. The significance of differences between different letters was assessed by Tukey’s HSD test (*P*<0.05).

**Table 7.** Correlation coefficients between investigation items in the field cultivation examination.

	SL	NN	SDW	NDN	PN	SN	100SW	Yi
SL	1.000							
NN	0.752***	1.000						
SDW	0.952***	0.708**	1.000					
NDN	0.763***	0.792***	0.634**	1.000				
PN	0.378	0.593*	0.308	0.647**	1.000			
SN	0.182	0.496	0.075	0.596*	0.861***	1.000		
100SW	0.680**	0.415	0.755***	0.432	0.325	–0.123	1.000	
Yi	0.618*	0.707**	0.578*	0.782***	0.930***	0.754***	0.553*	1.000

Asterisks denote significant differences at * *P*<0.05, ** *P*<0.01, and *** *P*<0.001. SL, NN, SDW, NDN, PN, SN, 100SW, and Yi represent shoot length, node number, shoot dry weight, nodule number, pod number, seed number, 100-seed weight, and yield, respectively.

**Table 8.** Occupancy rates (%) of bradyrhizobia infecting adzuki bean.

Cultivar	Treatment	Bj6	Bj123	Be31	Bd110	
					Hup^–^	Hup^+^
Iwate-dainagon	Control	0.0±0.0	0.0±0.0	90.1±6.1	9.9±6.1	0.0±0.0
	AMP1	0.0±0.0	0.0±0.0	80.2±8.6	6.6±3.3	13.2±7.6
	AMS5	0.0±0.0	0.0±0.0	86.5±2.0	0.0±0.0	13.5±2.0
	AN9	1.8±1.8	0.0±0.0	74.4±3.9	6.9±3.5	16.9±3.7
Hokuto-dainagon	Control	0.0±0.0	2.8±2.8	81.9±4.4	10.5±1.0	4.8±4.8
	AMP1	0.0±0.0	0.0±0.0	90.0±5.0	6.7±4.4	3.3±3.3
	AMS5	0.0±0.0	0.0±0.0	59.4±6.4	19.2±7.2	21.4±1.9
	AN9	0.0±0.0	0.0±0.0	80.8±7.4	4.4±2.4	14.8±8.3
Takara-shozu	Control	0.0±0.0	0.0±0.0	72.4±7.6	20.1±5.1	7.5±5.3
	AMP1	0.0±0.0	0.0±0.0	70.2±6.3	5.8±3.6	24.0±5.5
	AMS5	1.9±1.9	0.0±0.0	78.5±7.1	5.6±3.2	14.0±4.2
	AN9	1.4±1.4	0.0±0.0	75.6±9.1	9.7±7.7	13.3±2.6
Tanba-dainagon	Control	0.0±0.0	0.0±0.0	73.0±4.3	14.2±1.7	12.8±2.7
	AMP1	0.0±0.0	0.0±0.0	66.2±17.5	30.2±16.4	3.6±1.7
	AMS5	3.3±1.6	0.0±0.0	47.9±10.6	17.6±7.9	31.2±4.5
	AN9	4.2±2.4	0.0±0.0	88.7±6.9	0.0±0.0	7.1±5.0
Iwate-dainagon		0.4±0.4	0.0±0.0	82.9±3.0	5.8±2.0	10.9±2.7
Hokuto-dainagon		0.0±0.0	0.7±0.7	78.1±4.2	10.2±2.5	11.0±3.1
Takara-shozu		0.8±0.6	0.0±0.0	74.2±3.4	10.3±2.8	14.7±2.6
Tanba-dainagon		1.9±0.8	0.0±0.0	68.9±6.4	15.5±5.1	13.7±3.6
	Control	0.0±0.0	0.7±0.7	79.3±3.3	13.7±2.1	6.3±2.1 b
	AMP1	0.0±0.0	0.0±0.0	76.7±5.3	12.3±4.9	11.0±3.3 ab
	AMS5	1.3±0.7	0.0±0.0	68.1±5.5	10.6±3.4	20.0±2.6 a
	AN9	1.8±0.8	0.0±0.0	80.0±3.5	5.2±2.2	13.0±2.5 ab
ANOVA	Cultivar (C)	ns	ns	ns	ns	ns
	Treatment (T)	*	ns	ns	ns	**
	C×T	ns	ns	ns	ns	**

Isolates that showed similar RFLP patterns to *Bradyrhizobium japonicum* USDA 6^T^, USDA 123, *B. elkanii* USDA 31, and *B. diazoefficiens* USDA 110^T^ were designated as Bj6, Bj123, Be31, and Bd110, respectively. Regarding Bd110, strains possessing both the *hupL* and *hupS* genes were classified as Hup^+^ strains, while those lacking one or both genes were classified as Hup^–^ strains. Values represent the mean±standard error (*n*=3). Asterisks denote significant differences at * *P*<0.05, and ** *P*<0.01. The significance of differences between different letters was assessed by Tukey’s HSD test (*P*<0.05).

## References

[B1] Ayalew, T., and Yoseph, T. (2020) Symbiotic effectiveness of inoculation with *Bradyrhizobium* isolates on Cowpea (*Vigna unguiculata* (L.) Walp) varieties. Cogent Food Agric 6: 1845495.

[B2] Brignoli, D., Frickel-Critto, E., Sandobal, T.J., Balda, R.S., Castells, C.B., Mongiardini, E.J., et al. (2024) Quality control of *Bradyrhizobium* inoculant strains: detection of *nosZ* and correlation of symbiotic efficiency with soybean leaf chlo­rophyll levels. Front Agron 6: 1336433.

[B3] Champion, R.A., Mathis, J.N., Israel, D.W., and Hunt, P.G. (1992) Response of soybean to inoculation with efficient and inefficient *Bradyrhizobium japonicum* variants. Crop Sci 32: 457–463.

[B4] Chibeba, A.M., Kyei-Boahen, C., Guimarães, M.F., Nogueira, M.A., and Hungria, M. (2017) Isolation, characterization and selection of indigenous *Bradyrhizobium* strains with outstanding symbiotic performance to increase soybean yields in Mozambique. Agric Ecosyst Environ 246: 291–305. 28775390 10.1016/j.agee.2017.06.017 PMC5521954

[B5] Christopher, M., Macdonald, B., Yeates, S., Ziegler, D., and Seymour, N. (2018) Wild bradyrhizobia that occur in the Burdekin region of Queensland are as effective as commercial inoculum for mungbean (*Vigna radiata* (L.)) and black gram (*Vigna mungo* (L.)) in fixing nitrogen and dry matter production. Appl Soil Ecol 124: 88–94.

[B6] Delamuta, J.R., Ribeiro, R.A., Ormeño-Orrillo, E., Melo, I.S., Martínez-Romero, E., and Hungria, M. (2013) Polyphasic evidence supporting the reclassification of *Bradyrhizobium japonicum* group Ia strains as *Bradyrhizobium diazoefficiens* sp. nov. Int J Syst Evol Microbiol 63: 3342–3351. 23504968 10.1099/ijs.0.049130-0

[B7] Delić, D., Stajković, O., Rasulić, N., Kuzmanović, D., Jošić, D., and Miličić, B. (2010) Nodulation and N_2_ fixation effectiveness of *Bradyrhizobium* strains in symbiosis with adzuki bean, *Vigna angularis*. Braz Arch Biol Technol 53: 293–299.

[B8] Drevon, J.J., Kalia, V.C., Heckmann, M.O., and Salsac, L. (1987) Influence of the *Bradyrhizobium japonicum* hydrogenase on the growth of Glycine and Vigna species. Appl Environ Microbiol 53: 610–612. 16347309 10.1128/aem.53.3.610-612.1987 PMC203717

[B9] Evans, H.J., Harker, A.R., Papen, H., Russell, S.A., Hanus, F.J., and Zuber, M. (1987) Physiology, biochemistry, and genetics of the uptake hydrogenase in rhizobia. Annu Rev Microbiol 41: 335–361. 3318673 10.1146/annurev.mi.41.100187.002003

[B10] Fujihara, S., Terakado, J., and Nishibori, N. (2006) Accumulation of an aromatic amine, β-phenethylamine, in root nodules of adzuki bean *Vigna angularis*. Plant Soil 280: 229–237.

[B11] Goto, K., Takahashi, M., Nishiiri, K., and Abe, K. (1985) Effect of overhead flooding on growth and yield of soybean and azuki beans. Res Bull Hokkaido Natl Agric Exp Sta 141: 127–145 (in Japanese).

[B12] Gough, E.C., Owen, K.J., Zwart, R.S., and Thompson, J.P. (2021) Arbuscular mycorrhizal fungi acted synergistically with *Bradyrhizobium* sp. to improve nodulation, nitrogen fixation, plant growth and seed yield of mung bean (*Vigna radiata*) but increased the population density of the root-lesion nematode *Pratylenchus thornei*. Plant Soil 465: 431–452.

[B13] Hayashi, M., Saeki, Y., Haga, M., Harada, K., Kouchi, H., and Umehara, Y. (2012) *Rj* (*rj*) genes involved in nitrogen-fixing root nodule formation in soybean. Breed Sci 61: 544–553. 23136493 10.1270/jsbbs.61.544 PMC3406786

[B14] Hiraishi, A., Kamagata, Y., and Nakamura, K. (1995) Polymerase chain reaction amplification and restriction fragment length polymorphism ana­lysis of 16S rRNA genes from methanogens. J Ferment Bioeng 79: 523–529.

[B15] Hu, L., Luo, G., Zhu, X., Wang, S., Wang, L., Cheng, X., and Chen, H. (2022) Genetic diversity and environmental influence on yield and yield-related traits of adzuki bean (*Vigna angularis* L.). Plants 11: 1132–1145. 35567132 10.3390/plants11091132 PMC9103669

[B16] Hungria, M., Boddey, L.H., Santos, M.A., and Vargas, M.A.T. (1998) Nitrogen fixation capacity and nodule occupancy by *Bradyrhizobium japonicum* and *B. elkanii* strains. Biol Fertil Soils 27: 393–399.

[B17] Iseki, R. (2021) The page of Iseki Ryuta. ANOVAKUN (in Japanese). URL https://riseki.cloudfree.jp/?ANOVA君

[B18] Kimura, S.D., Schmidtke, K., Tajima, R., Yoshida, K., Nakashima, H., and Rauber, R. (2004) Seasonal N uptake and N_2_ fixation by common and adzuki bean at various spacings. Plant Soil 258: 91–101.

[B19] Leng, P., Jin, F., Li, S., Huang, Y., Zhang, C., Shan, Z., et al. (2023) High efficient broad-spectrum *Bradyrhizobium elkanii* Y63-1. Oil Crop Sci 8: 228–235.

[B20] Mason, M.L.T., Matsuura, S., Domingo, A.L., Yamamoto, A., Shiro, S., Sameshima-Saito, R., and Saeki, Y. (2017) Genetic diversity of indigenous soybean-nodulating *Bradyrhizobium elkanii* from southern Japan and Nueva Ecija, Philippines. Plant Soil 417: 349–362.

[B21] Mason, M.L.T., De Guzman, B.L.T., Yamamoto, A., and Saeki, Y. (2021) Symbiotic performance of indigenous soybean bradyrhizobia from the Philippines with soybean (*Glycine max* [L.] Merill) cultivars harboring different *Rj* genotypes. Symbiosis 83: 55–63.

[B22] Masuda, S., Saito, M., Sugawara, C., Itakura, M., Eda, S., and Minamisawa, K. (2016) Identification of the hydrogen uptake gene cluster for chemolithoautotrophic growth and symbiosis hydrogen uptake in *Bradyrhizobium diazoefficiens*. Microbes Environ 31: 76–78. 26911707 10.1264/jsme2.ME15182 PMC4791120

[B23] Minami, M., Yamakawa, T., Yamamoto, A., Akao, S., and Saeki, Y. (2009) Estimation of nodulation tendency among *Rj*-genotype soybeans using the bradyrhizobial community isolated from an andosol. Soil Sci Plant Nutr 55: 65–72.

[B24] Ministry of Agriculture, Forestry and Fisheries, Japan. (2024) Harvest of adzuki beans, green beans, and ground nuts (dried grain) in 2023 (in Japanese). URL https://www.maff.go.jp/j/tokei/kekka_gaiyou/tokutei_sakumotu/r5/syukaku_mame/index.html

[B25] Nguyen, H.P., Miwa, H., Kaneko, T., Sato, S., and Okazaki, S. (2017) Identification of *Bradyrhizobium elkanii* genes involved in incompatibility with *Vigna radiata*. Genes 8: 374. 29292795 10.3390/genes8120374 PMC5748692

[B26] Nguyen, H.P., Ratu, S.T.N., Yasuda, M., Teaumroong, N., and Okazaki, S. (2020) Identification of *Bradyrhizobium elkanii* USDA61 type III effectors determining symbiosis with *Vigna mungo*. Genes 11: 474. 32349348 10.3390/genes11050474 PMC7291247

[B27] Pereira, S., Singh, S., Oliveira, R.S., Ferreira, L., Rosa, E., and Marques, G. (2020) Co-inoculation with rhizobia and mycorrhizal fungi increases yield and crude protein content of cowpea (*Vigna unguiculata* (L.) Walp.) under drought stress. J Sustainable Organic Agric Syst 70: 56–65.

[B28] R Development Core Team. (2022) R: A Language and Environment for Statistical Computing, Vienna, Austria, R Foundation for Statistical Computing. URL https://www.r-project.org

[B29] Saeki, Y., Aimi, N., Hashimoto, M., Tsukamoto, S., Kaneko, A., Yoshida, N., et al. (2004) Grouping of *Bradyrhizobium* USDA strains by sequence ana­lysis of 16S rDNA and 16S-23S rDNA internal transcribed spacer region. Soil Sci Plant Nutr 50: 517–525.

[B46] Saeki, Y., Aimi, N., Tsukamoto, S., Yamakawa, T., Nagatomo, Y., and Akao, S. (2006) Diversity and geographical distribution of indigenous soybean-nodulating bradyrhizobia in Japan. Soil Sci Plant Nutr 52: 418–426.

[B30] Saeki, Y., Nakamura, M., Mason, M.L.T., Yano, T., Shiro, S., Sameshima-Saito, R., et al. (2017) Effect of flooding and the *nosZ* gene in bradyrhizobia on bradyrhizobial community structure in the soil. Microbes Environ 32: 154–163. 28592720 10.1264/jsme2.ME16132 PMC5478539

[B31] Sameshima-Saito, R., Chiba, K., and Minamisawa, K. (2006) Correlation of denitrifying capability with the existence of *nap*, *nir*, *nor* and *nos* genes in diverse strains of soybean bradyrhizobia. Microbes Environ 21: 174–184.

[B32] Sato, H., Matsukawa, I., Narikawa, T., and Ushirogi, T. (1975) The new adzuki bean variety “Sakae-shozu”. Bull Hokkaido Prefect Agric Exp Stn 33: 58–67 (in Japanese).

[B33] Shiina, Y., Itakura, M., Choi, H., Saeki, Y., Hayatsu, M., and Minamisawa, K. (2014) Relationship between soil type and N_2_O reductase genotype (*nosZ*) of indigenous soybean bradyrhizobia: *nosZ*-minus populations are dominant in andosols. Microbes Environ 29: 420–426. 25476067 10.1264/jsme2.ME14130 PMC4262367

[B34] Shimada, H., Murata, K., Fujita, S., Chiba, I., Hara, M., Shirai, S., and Adachi, T. (1997) A new adzuki bean variety “Hokuto-dainagon”. Bull Hokkaido Prefect Agric Exp Stn 72: 85–95 (in Japanese).

[B35] Shiro, S., Yamamoto, A., Umehara, Y., Hayashi, M., Yoshida, N., Nishiwaki, A., et al. (2012) Effect of *Rj* genotype and cultivation temperature on the community structure of soybean-nodulating bradyrhizobia. Appl Environ Microbiol 78: 1243–1250. 22156423 10.1128/AEM.06239-11 PMC3272988

[B36] Shiro, S., Kuranaga, C., Yamamoto, A., Sameshima-Saito, R., and Saeki, Y. (2016) Temperature-dependent expression of *nodC* and community structure of soybean-nodulating bradyrhizobia. Microbes Environ 31: 27–32. 26877137 10.1264/jsme2.ME15114 PMC4791112

[B37] Shiro, S., and Saeki, Y. (2022) Breeding of *Rj* gene-accumulated soybean genotypes and their availability for improving soybean productivity. In *Soybean – Recent Advances in Research and Applications*. Ohyama, T., Takahashi, Y., Ohtake, N., Sato, T., and Tanabata, S. (eds). London: IntechOpen, pp. 159–178.

[B38] Shiro, S., Makihara, R., Yamaguchi, M., Kadowaki, M., and Saeki, Y. (2023) Compatibility of adzuki bean (*Vigna angularis*) and *Bradyrhizobium* USDA strains, and geographical distribution and community structure on indigenous adzuki bean-nodulating bradyrhizobia in Japan. Plant Prot Sci 59: 217–232.

[B39] Soe, K.M., Bhromsiri, A., Karladee, D., and Yamakawa, T. (2012) Effects of endophytic actinomycetes and *Bradyrhizobium japonicum* strains on growth, nodulation, nitrogen fixation and seed weight of different soybean varieties. Soil Sci Plant Nutr 58: 319–325.

[B40] Ushio, A., Koroda, Y., Isono, T., Fujita, K., and Matsumoto, I. (2013) Characteristics of adzuki bean “Tanbadainagon” in narrow row and dense seeding cultivation. Bull Hyogo Prefect Technol Cent Agric For Fish Agric Sect 61: 33–35 (in Japanese).

[B41] Vincent, J.M. (1970) *A Manual for the Practical Study of the Root-Nodule Bacteria*. (*International Biological Programme*). Oxford: Blackwell Scientific Publications.

[B42] Vlassak, K.M., Vanderleyden, J., and Graham, P.H. (1997) Factors influencing nodule occupancy by inoculant rhizobia. Crit Rev Plant Sci 16: 163–229.

[B43] Xiao, G. (2024) Dry bean production and distribution in China’s major producing areas: Focus on Heilongjiang Province and inner Mongolia autonomous region. Economia 74: 53–69 (in Japanese).

[B47] Yin, Z., Guo, W., Liang, J., Xiao, H., Hao, X., Hou, A., et al. (2019) Effect of multiple N, P, and K fertilizer combinations on adzuki bean (Vigna angularis) yield in a semi-arid region of northeastern China. Sci Rep 9: 19408. 31857646 10.1038/s41598-019-55997-9 PMC6923502

[B44] Yoneda, A., Hurusawa, N., Sasaki, K., Murakami, T., Sato, T., Ono, Y., et al. (1973) Adzuki bean variety “Iwate-dainagon”. Tohoku J Agric Res 14: 188–192 (in Japanese).

[B45] Yuhashi, K., Akao, S., Fukuhara, H., Tateno, E., Chun, J.Y., Stacey, G., et al. (1995) *Bradyrhizobium elkanii* induces outer cortical root swelling in soybean. Plant Cell Physiol 36: 1571–1577.

